# Supplementary material for: The “Crosstalk” between Microbiota and Metabolomic Profile of Kefalograviera Cheese after the Innovative Feeding Strategy of Dairy Sheep by Omega-3 Fatty Acids
Source: Foods. 2022 Oct 11;11(20):3164. doi: 10.3390/foods11203164 (PMC9601511; doi:10.3390/foods11203164)
Supplement: Supplementary file 1 [file foods-11-03164-s001.zip › foods-1937010-supplementary.pdf]

foods MDPI

Table S1. Daily ingredient allowance and chemical composition of the diets offered to dairy ewes during the trial.

Ingredients (on fresh weight basis)	Control diet (g/day/ewe)	Experimental diet (g/day/ewe)
Lucerne hay	1.200	1.200
Barley straw	300	300
Corn grain	540	590
Barley grain	330	150
Wheat bran	180	110
Sunflower seed meal (36% crude protein)	30	180
Soyabean meal (47% crude protein)	300	150
Cotton seed	30	80
Flaxseed	-	75
Lupin seed	-	75
Molasses	20	20
Premix[1] with vitamins and inorganic minerals	60	60
Total dry matter intake / day	2630	2640
**Chemical Analysis (%)**		
Dry matter	87.4	87.5
Crude protein (N x 6.25)	16.1	16.1
Ether extract	2.82	4.01
Crude fibre	16.6	17.7
Neutral detergent fibre	33.8	35.1
Acid detergent fibre	19.5	19.9
Acid detergent lignin	4.51	4.98
Ash	6.55	6.52
Starch	18	16.1

Sugars	5.11	5.04
**Calculated analysis**		
Calcium (g/kg)	11.5	11.6
Phosphorus (total) (g/kg)	3.82	3.98
PDI (g/kg DM)	89.4	87.5
PDIA (g/kg DM)	44.3	42.7
UF_L	0.71	0.71
Total saturated fatty acids (%)	0.48	0.59
Total unsaturated fatty acids (%)	1.69	2.72
Omega-6 fatty acids (%)	0.99	1.15
Omega-3 fatty acids (%)	0.28	0.82
Omega-6 / Omega-3	3.53	1.41

[1]Vitamin and mineral mix contained per kg DM of concentrate: 8,000 IU of vitamin A; 90 mg of vitamin E; 3,000 IU of vitamin D_3; 1.5 mg/kg biotin; 6 mg/kg niacin; 45 mg/kg choline; 0,2 mg Co; 3 mg I; 100 mg/kg Fe; 50 mg Mn; 0,45 mg Se; 150 mg Zn; 6 g of NaCl; 4 g of sulphur; 10 g of magnesium oxide; 15 g of monocalcium phosphate and 21 g of limestone. PDI = protein digestible in the small intestine, PDIA= protein digestible in the small intestine supplied by rumen –undegraded dietary protein, UF_L= forage unit for lactation.

Table S2. List of compounds detected and identified in identification level 1.

Compound name	Category	Chromatography	Ionization
γ-Aminobutyric acid	gamma-amino acid	HILIC, RPLC	positive
(R)-(+)-2-Pyrrolidone-5-carboxylic acid	amino acid derivative	HILIC	positive
(RS)-Mevalonic acid lithium salt	dihydroxy monocarboxylic acid	HILIC, RPLC	negative
10-Hydroxydecanoic acid	fatty acid	RPLC	positive
1-Aminocyclopropanecarboxylic acid	non-proteinogenic alpha-amino acid	RPLC	positive
1-Methyladenosine	purine nucleosides	HILIC	positive
1-Methyl-L-histidine	amino acid derivative	HILIC	positive
2-Aminoisobutyric acid	non-protein amino acid	HILIC, RPLC	positive
2-Aminophenol	aminophenol	HILIC	positive
2-Oxoadipic acid	dicarboxylic organic acid	RPLC	negative
3-(2-Hydroxyethyl)indole	indolyl alcohol	RPLC	positive
3-Amino-4-hydroxybenzoic acid	monohydroxybenzoic acid	HILIC, RPLC	positive
3-amino-5-hydroxybenzoic acid	monohydroxybenzoic acid	HILIC	positive
3-Dehydroshikimic acid	monocarboxylic organic acid	RPLC	negative
3-Hydroxy-3-methylglutaric acid	dicarboxylic organic acid	RPLC	positive
3-Hydroxybenzyl alcohol	benzyl alcohol	HILIC	negative
3-Methoxytyramine hydrochloride	catecholamine neurotransmitter / extracellular metabolite of dopamine	HILIC	positive
3-Methyl-2-oxindole	oxindoles	RPLC, HILIC	positive
3-Methyladenine	adenine substituted with a methyl group	HILIC	positive
3-Ureidopropionic acid	conjugate acid of a N-carbamoyl-beta-alaninate	HILIC	positive
4-Acetamidobutyric Acid	acetamides	HILIC, RPLC	positive
4-Coumarate	conjugate base of 4-coumaric acid	RPLC	negative
4-Guanidinobutyric acid	gamma amino acid derivative	HILIC	positive
4-Hydroxybenzaldehyde	benzaldehyde	HILIC, RPLC	negative
4-Hydroxybenzoic acid	monohydroxybenzoic acid	HILIC	negative
4-Imidazoleacetic acid hydrochloride	acetic acid	RPLC	positive
4-Imidazoleacrylic acid	monocarboxylic organic acid	RPLC	positive
4-Pyridoxic acid	methylpyridine	HILIC	positive
5-Aminovaleric acid	delta-amino acid	HILIC	positive
5-Hydroxyindole-3-acetic acid	indole-3-acetic acids	RPLC	positive
5-Hydroxy-L-tryptophan	L-alpha-amino acid	RPLC	positive
6-(γ,γ-Dimethylallylamino)purine	precursor of cytokinin	HILIC	positive
Adenine	purine nucleobase	HILIC	positive
Adipic acid	omega-dicarboxylic organic acid	RPLC	positive
Allantoin	urea	RPLC	positive
Arabinose	monosaccharide	RPLC, HILIC	positive
Azelaic acid	dicarboxylic organic acid	RPLC	positive
Betaine	amino acid	HILIC	positive
Biotin	vitamin B_7	HILIC	positive
Bis(2-Ethylhexyl)Phthalate	diester of phthalic acid	RPLC	positive
cis-4-Hydroxy-D-proline	amino acid derivative	HILIC	positive
Citric acid	tricarboxylic organic acid	RPLC	negative
Creatine	amino acid derivative	HILIC, RPLC	positive
Cytosine	pyrimidine base	HILIC	positive
d-Desthiobiotin	vitamin B_7 derivative	HILIC	positive
Decanoate	fatty acid	RPLC	negative
Dihydrouracil	pyrimidine	HILIC	positive
DL-2-Aminoadipic acid	amino acid	RPLC	positive
DL-Normetanephrine hydrochloride	metabolite of Epinephrine	HILIC	positive
DL-p-Hydroxyphenyllactic acid	carboxylic organic acid	HILIC	negative
Dulcitol	sugar alcohol	RPLC	negative
Elaidate	fatty acid	RPLC	negative

Compound name	Category	Chromatography	Ionization
Ethylmalonic acid	dicarboxylic organic acid	RPLC	negative
Glycerol-Myristate	1-monoglyceride of myristic acid	RPLC	positive
Guanosine 3',5'-cyclic monophosphate sodium salt	Cyclic nucleotide	HILIC	positive
Heptadecanoate	fatty acid	RPLC	positive
Hypoxanthine	purine-based organic compound	HILIC	negative
Itaconic acid	dicarboxylic organic acid	RPLC	negative
L-Alanine	amino acid	HILIC, RPLC	positive
L-Arginine monohydrochloride	hydrochloride salt of arginine	HILIC	positive
L-Ascorbic acid	Vitamin C	RPLC	positive
L-Asparagine	amino acid	HILIC, RPLC	positive
L-Carnitine hydrochloride	Vitamin B_T	HILIC, RPLC	positive
L-Citrulline	amino acid	HILIC	positive
L-Glutamic acid	amino acid	HILIC, RPLC	positive
L-Glutamine	amino acid	RPLC, HILIC	positive
L-Homoserine	amino acid	HILIC, RPLC	positive
L-Isoleucine	amino acid	HILIC, RPLC	positive
L-Methionine	amino acid	HILIC, RPLC	positive
L-Norleucine	amino acid	HILIC, RPLC	positive
L-Norvaline	amino acid	HILIC	positive
L-Ornithine monohydrochloride	polyamine	HILIC, RPLC	positive
L-Phenylalanine	amino acid	HILIC, RPLC	positive
L-Proline	amino acid	HILIC	positive
L-Pyroglutamic acid	amino acid derivative	HILIC, RPLC	positive
L-Serine	amino acid	HILIC	positive
L-Threonine	amino acid	HILIC	positive
Lumichrome	flourescent photoproduct of riboflavin degradation	RPLC, HILIC	positive
L-Valine	amino acid	HILIC, RPLC	positive
Maleic acid	dicarboxylic organic acid	RPLC	negative
Malonic acid	omega-dicarboxylic organic acid	RPLC	negative
Methyl 4-aminobutyrate hydrochloride	methyl ester	HILIC	positive
Methyl indole-3-acetate	methyl ester	RPLC	positive
Methylmalonic acid	dicarboxylic organic acid	RPLC	negative
Myristate	fatty acid	RPLC	negative
N-Acetyl-5-hydroxytryptamine	acetamides	HILIC	positive
N-Acetyl-D-glucosamine	amide derivative	RPLC	positive
N-Acetyl-DL-methionine	N-acetyl-amino acid	RPLC	positive
N-Acetyl-DL-serine	N-acetyl-amino acid	HILIC, RPLC	negative
N-Acetyl-L-alanine	N-acetyl-amino acid	HILIC	positive
N-Acetyl-L-leucine	N-acetyl-amino acid	RPLC, HILIC	negative
N-Acetyl-L-phenylalanine	N-acetyl-amino acid	RPLC, HILIC	negative
N-Acetyl-L-proline	N-acetyl-amino acid	RPLC	positive
N-Formyl-L-methionine	amino acid derivative	RPLC	positive
Nicotinamide	form of Vitamin B_3	HILIC	positive
Nicotinic acid	Vitamin B_3	HILIC	positive
N-Methyl-D-aspartic acid	amino acid derivative	HILIC, RPLC	positive
N-Methyl-L-glutamic acid	amino acid derivative	RPLC	positive
Nα-Acetyl-L-lysine	N-acetyl-amino acid	RPLC	positive
Oleate	fatty acid	RPLC	negative
Omega-Hydroxydodecanoate	fatty acid	RPLC, HILIC	negative
O-Phosphorylethanolamine	monophosphate ethanolamine ester	RPLC	positive
O-Succinyl-L-homoserine	amino acid derivative	RPLC	positive
Palmitate	fatty acid	RPLC	negative
Petroselinate	fatty acid	RPLC	negative
Pimelic acid	omega-dicarboxylic organic acid	RPLC	positive
Purine	heterocyclic aromatic organic compound	HILIC	positive
Pyridoxal 5'-phosphate hydrate	active form of vitamin B_6	RPLC	positive
Pyridoxamine dihydrochloride	vitamin B_6 derivative	HILIC	positive
Salicylamide	salicylamides	HILIC	positive
Sarcosine	amino acid derivative	HILIC, RPLC	positive
Sphinganine	aminodiol	RPLC	positive
Stearate	fatty acid	RPLC	negative
Succinic acid	omega-dicarboxylic organic acid	RPLC, HILIC	negative
Theobromine	alkaloid	RPLC	positive
Thymine	pyrimidine nucleobase	HILIC	positive
Trigonelline hydrochloride	alkaloid	HILIC, RPLC	positive
Tryptamine	aminoalkylindole	RPLC	positive
Tyramine	monoamine compound	RPLC, HILIC	positive
Uracil	pyrimidine nucleobase	RPLC, HILIC	positive
Uric acid	organic acid	RPLC	negative
Xanthine	oxopurine	HILIC	negative

The acceptable criteria used for the identification of the detected compounds were retention time (±0.2 min related to the analytical standard), mass accuracy (mass error ≤ 2 mDa), isotopic fitting (mSigma ≤ 50), MS/MS qualifier ions, peak area threshold >800 and intensity threshold >200.

The following compounds were identified in identification level 3, as the MS/MS spectra were not informative enough to proceed in further identification:

(RS)-Mevalonic acid lithium salt, 1-Methyladenosine, 1-Methyl-L-histidine, 2-Oxoadipic acid, 3-Dehydroshikimic acid, 3-Hydroxybenzyl alcohol, 3-Methyl-2-oxindole, 3-Methyladenine, 4-Acetamidobutyric acid, Adipic acid, Biotin, Citric acid, Dihydrouracil, DL-Normetanephrine hydrochloride, DL-p-Hydroxyphenyllactic acid, Dulcitol, Elaidate, Guanosine 3',5'-cyclic monophosphate sodium salt, Heptadecanoate, Itaconic acid, L-Ascorbic acid, Malonic acid, N-Acetyl-L-phenylalanine, N-Acetyl-L-proline, N-Methyl-L-glutamic acid, Oleate, O-Phosphorylethanolamine, O-Succinyl-L-homoserine, Palmitate, Petroselinate, Pimelic acid, Pyridoxal 5'-phosphate hydrate, Salicylamide, Sphinganine, Stearate, Theobromine, Uric acid.

Table S3. In-house database information for selected important metabolites.

Compound	Chromatography	Ionization	Retention time (min)	Precursor ion	Adduct of precursor ion	Fragments
L-Glutamine	RPLC, HILIC	positive	1.3, 7.6	147.0764	$[M+H]^+$	67.0544/84.0818/130.0878
L-Homoserine	RPLC, HILIC	positive	1.4, 7.5	120.0655	$[M+H]^+$	56.0492/74.06
L-Proline	HILIC	positive	6.5	116.0706	$[M+H]^+$	55.0538/70.0647/72.0804
Succinic acid	RPLC, HILIC	negative	1.2, 7.6	117.0193	$[M-H]^-$	59.0152/73.0304
Tryptamine	RPLC	positive	3.9	161.1073	$[M+H]^+$	71.0496/ 144.0808
Uracil	RPLC, HILIC	positive	1.6, 2.8	113.0346	$[M+H]^+$	87.027/61.0105/70.0655
L-Ornithine	RPLC, HILIC	positive	1.2, 9.5	133.0972	$[M+H]^+$	70.0647/116.0784

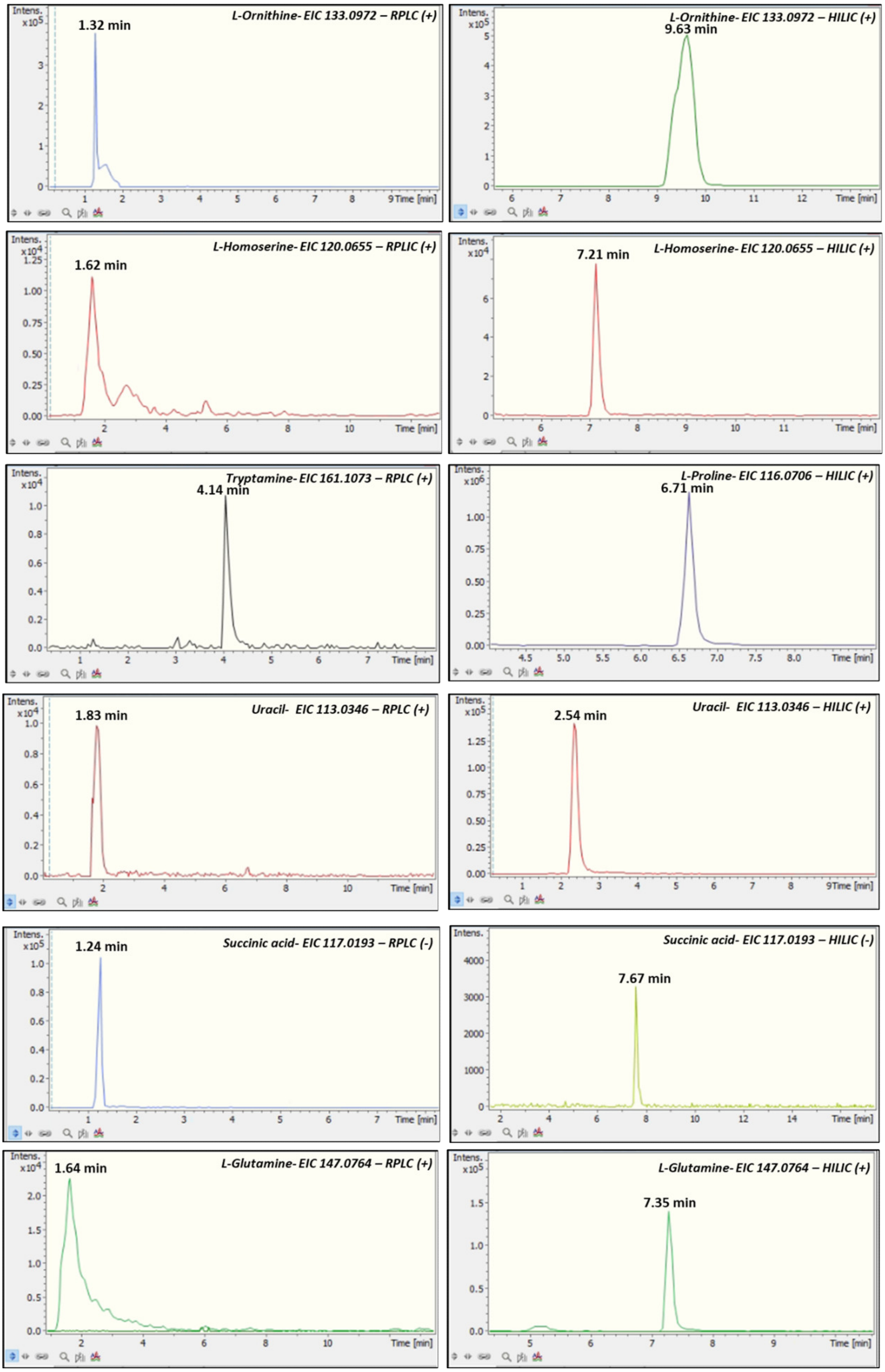

Figure S1. Extracted Ion Chromatograms (EIC) in quality control (QC) samples for selected important metabolites.

Quantification of the analytes was performed through matrix-matched standard calibration curve, at a range of 0.2 μg/g – 20 μg/g. The matrix-matched samples contained metabolite sub-mixtures, the sample extract (blank without IS) and appropriate volume of Internal Standard (IS). The initial standard solutions were prepared according to producers' guidelines (https://www.iroatech.com/mass-spectrometry-metabolite-library-ofstandards-msmls/). Internal Standards were used to evaluate the instrumental performance through the analytical batch. Taking into account the pre-concentration factor through the sample preparation procedure (2x) concentrations were calculated of each metabolite, for each lyophilized cheese sample. For the calculation of real concentrations, using weights of the lyophilized and fresh cheese samples (calculating the loss of water), we provided the concentration (μg/g) of the metabolites in the initial fresh cheese samples. Samples were classified in groups (Group 1 - control diet and Group 2- Experimental diet) and calculated the average concentrations (μg/g) of each metabolite, in each group. The results represented in Fig. S1.

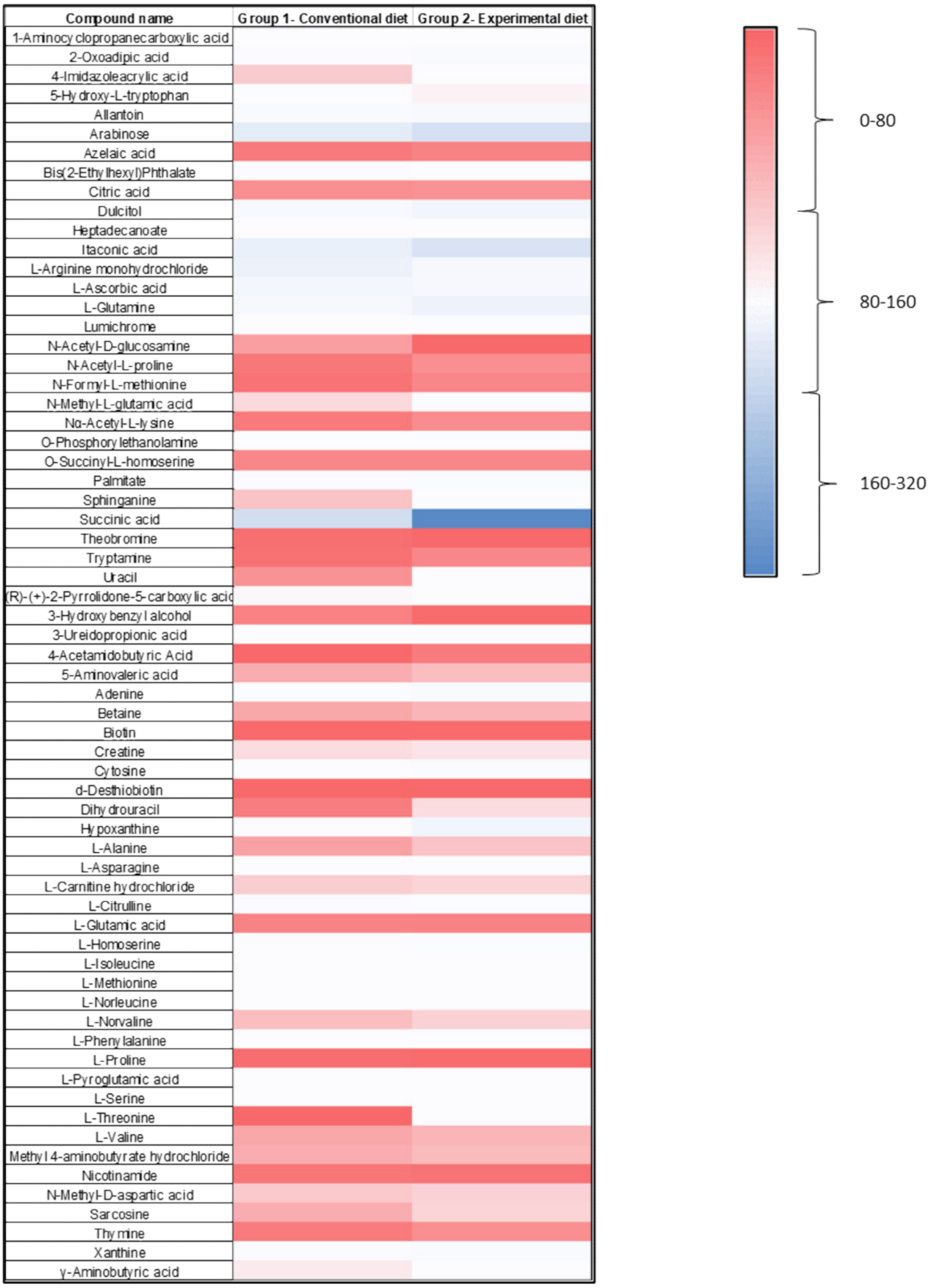

Figure S2. Heat map for selected quantified metabolites (in μg/g) for the studied groups.

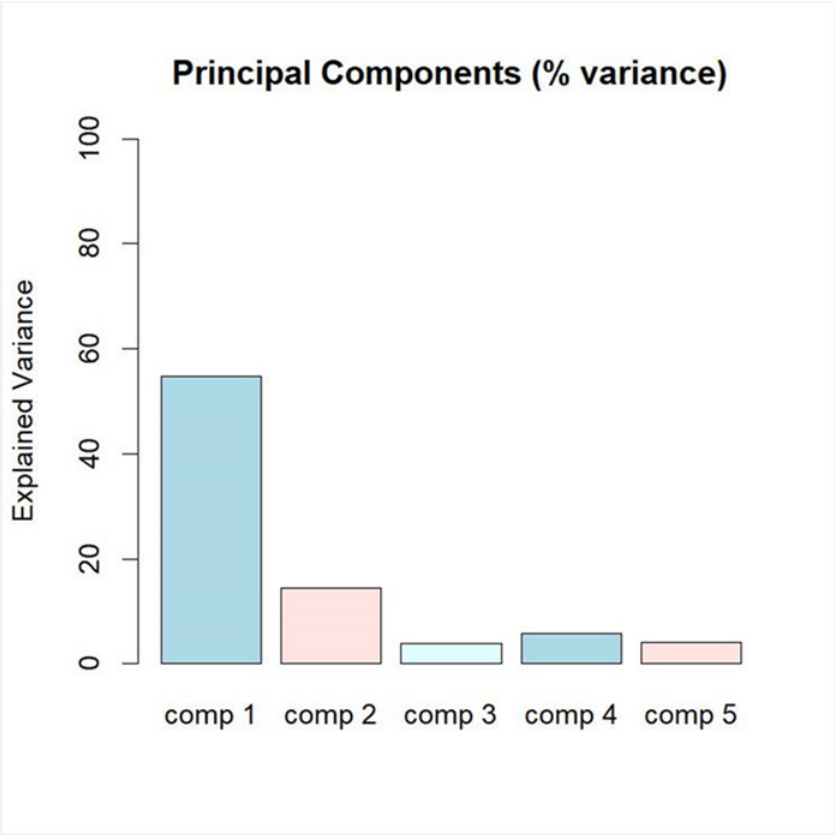

Figure S3. Evaluation of Principal Components (PCs) importance in PCA.
